# 4,5,6,7-Tetra­chloro-*N*-(2,3,4-trifluoro­phen­yl)phthalimide

**DOI:** 10.1107/S1600536811020721

**Published:** 2011-06-11

**Authors:** Yin-Jun Zhang, Chen-Tao Luo, Yu-Guang Wang, Zhao Wang

**Affiliations:** a College of Biological and Environmental Engineering, Zhejiang University of Technology, Hangzhou 310032, People’s Republic of China

## Abstract

The asymmetric unit of the title compound, C_14_H_2_Cl_4_F_3_NO_2_, contains two independent mol­ecules. In each mol­ecule, the phthalimide ring system is nearly planar [maximum atomic deviation = 0.031 (2) or 0.038 (2) Å] and oriented with respect to the benzene ring at 65.04 (7) or 71.76 (10)°. Weak inter­molecular C—H⋯O and C—H⋯F hydrogen bonding is present in the crystal structure.

## Related literature

For the title compound as an inter­mediate of organic electro-luminescent materials, see: Han & Kay (2005 ▶). For the synthesis, see: Valkonen *et al.* (2007 ▶); Barchin *et al.* (2002 ▶). For related structures, see: Xu *et al.* (2006 ▶); Fu *et al.* (2010*a* ▶,*b* ▶,*c* ▶).
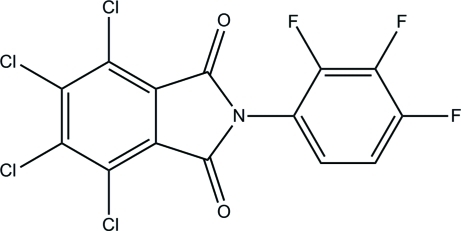


## Experimental

### 

#### Crystal data

C_14_H_2_Cl_4_F_3_NO_2_
*M* *_r_* = 414.97
Triclinic, 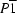

*a* = 6.7722 (7) Å
*b* = 8.8052 (12) Å
*c* = 24.493 (3) Å
α = 95.777 (9)°
β = 94.514 (17)°
γ = 98.839 (12)°
*V* = 1429.2 (3) Å^3^
*Z* = 4
Mo *K*α radiation
μ = 0.87 mm^−1^
*T* = 113 K
0.20 × 0.18 × 0.12 mm

#### Data collection

Rigaku Saturn 724CCD area-detector diffractometer
Absorption correction: multi-scan (*SADABS*; Bruker, 2001 ▶) *T*_min_ = 0.845, *T*_max_ = 0.903
13490 measured reflections
6684 independent reflections
4832 reflections with *I* > 2σ(*I*)
*R*_int_ = 0.033

#### Refinement

*R*[*F* ^2^ > 2σ(*F* ^2^)] = 0.038
*wR*(*F* ^2^) = 0.095
*S* = 0.98
6684 reflections
433 parameters
H-atom parameters constrained
Δρ_max_ = 0.62 e Å^−3^
Δρ_min_ = −0.38 e Å^−3^

### 

Data collection: *SMART* (Bruker, 2007 ▶); cell refinement: *SAINT* (Bruker, 2007 ▶); data reduction: *SAINT*; program(s) used to solve structure: *SHELXTL* (Sheldrick, 2008 ▶); program(s) used to refine structure: *SHELXTL*; molecular graphics: *SHELXTL*; software used to prepare material for publication: *SHELXTL*.

## Supplementary Material

Crystal structure: contains datablock(s) I, global. DOI: 10.1107/S1600536811020721/xu5228sup1.cif

Structure factors: contains datablock(s) I. DOI: 10.1107/S1600536811020721/xu5228Isup2.hkl

Supplementary material file. DOI: 10.1107/S1600536811020721/xu5228Isup3.cml

Additional supplementary materials:  crystallographic information; 3D view; checkCIF report

## Figures and Tables

**Table 1:** Hydrogen-bond geometry (Å, °)

*D*—H⋯*A*	*D*—H	H⋯*A*	*D*⋯*A*	*D*—H⋯*A*
C14—H14⋯O3^i^	0.95	2.44	3.210 (3)	138
C27—H27⋯F3^ii^	0.95	2.45	3.379 (3)	165
C28—H28⋯O1	0.95	2.50	3.403 (3)	158

Symmetry codes: (i) 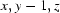; (ii) 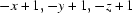.

**Fig. 1.**
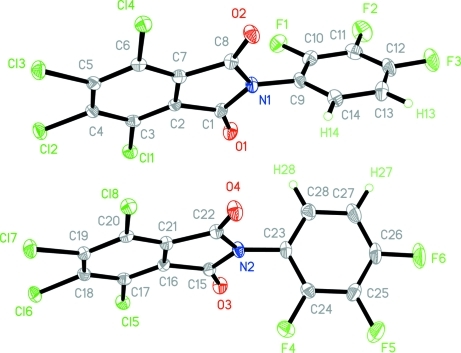
View of the molecule of showing the atom-labelling scheme. Displacement ellipsoids are drawn at the 30% probability level.

## supplementary crystallographic information

### Comment

The title compound is a key intermediate of organic electro-luminescent, the emission of light by organic molecules exposed in the electric field, which has been wide investigated in academic and industrial displays (Han & Kay, 2005).

The molecular structure of the title compound is illustrated in Fig. 1. In the title compound, two rings are nearly planar, but the molecule as a whole is not planar. The dihedral angle between the benzene ring and the phthalimide plane is 66.03 (12) °, which is greater than 60.3 (5)° found in a related compound 2-(2-iodoethyl)isoindole-1,3-dione (Valkonen *et al.* 2007). Intermolecular weak C—H···O and C—H···F hydrogen bonding is present in the crystal structure (Table 1).

### Experimental

Compound (I) was prepared according to the procedure in the literature (Barchin *et al.*, 2002). The reaction was initiated by the addition of one molar equivalent of 2,3,4-trifluoroaniline and one molar equivalents of 4,5,6,7-tetrachlorophthalic anhydride and subsequent refluxing overnight, and then filtered. The crude produce was washed with water three times, and dried. The compound resulted (yield 96%) and single crystals suitable for X-ray analysis were obtained by slow evaporation of a ethyl acetate solution.

### Refinement

H atoms were positioned geometrically and refined as riding with C—H = 0.95 Å, and *U*_iso_(H) = 1.2*U*_eq_(C).

### Crystal data

**Table:**

C_14_H_2_Cl_4_F_3_NO_2_	*Z* = 4
*M**_r_* = 414.97	*F*(000) = 816
Triclinic, *P*1	*D*_x_ = 1.928 Mg m^−^^3^
Hall symbol: -P 1	Mo *K*α radiation, λ = 0.71073 Å
*a* = 6.7722 (7) Å	Cell parameters from 4943 reflections
*b* = 8.8052 (12) Å	θ = 1.7–27.9°
*c* = 24.493 (3) Å	µ = 0.87 mm^−^^1^
α = 95.777 (9)°	*T* = 113 K
β = 94.514 (17)°	Prism, colorless
γ = 98.839 (12)°	0.20 × 0.18 × 0.12 mm
*V* = 1429.2 (3) Å^3^	

### Data collection

**Table:**

Rigaku Saturn 724CCD area-detector diffractometer	6684 independent reflections
Radiation source: rotating anode	4832 reflections with *I* > 2σ(*I*)
multilayer	*R*_int_ = 0.033
Detector resolution: 14.22 pixels mm^-1^	θ_max_ = 27.9°, θ_min_ = 1.7°
ω and φ scans	*h* = −8→8
Absorption correction: multi-scan (*SADABS*; Bruker, 2001)	*k* = −11→11
*T*_min_ = 0.845, *T*_max_ = 0.903	*l* = −31→32
13490 measured reflections	

### Refinement

**Table:**

Refinement on *F*^2^	Primary atom site location: structure-invariant direct methods
Least-squares matrix: full	Secondary atom site location: difference Fourier map
*R*[*F*^2^ > 2σ(*F*^2^)] = 0.038	Hydrogen site location: inferred from neighbouring sites
*wR*(*F*^2^) = 0.095	H-atom parameters constrained
*S* = 0.98	*w* = 1/[σ^2^(*F*_o_^2^) + (0.0384*P*)^2^] where *P* = (*F*_o_^2^ + 2*F*_c_^2^)/3
6684 reflections	(Δ/σ)_max_ = 0.001
433 parameters	Δρ_max_ = 0.62 e Å^−^^3^
0 restraints	Δρ_min_ = −0.38 e Å^−^^3^

### Special details

**Table:**

Geometry. All e.s.d.'s (except the e.s.d. in the dihedral angle between two l.s. planes) are estimated using the full covariance matrix. The cell e.s.d.'s are taken into account individually in the estimation of e.s.d.'s in distances, angles and torsion angles; correlations between e.s.d.'s in cell parameters are only used when they are defined by crystal symmetry. An approximate (isotropic) treatment of cell e.s.d.'s is used for estimating e.s.d.'s involving l.s. planes.
Refinement. Refinement of *F*^2^ against ALL reflections. The weighted *R*-factor *wR* and goodness of fit *S* are based on *F*^2^, conventional *R*-factors *R* are based on *F*, with *F* set to zero for negative *F*^2^. The threshold expression of *F*^2^ > σ(*F*^2^) is used only for calculating *R*-factors(gt) *etc*. and is not relevant to the choice of reflections for refinement. *R*-factors based on *F*^2^ are statistically about twice as large as those based on *F*, and *R*- factors based on ALL data will be even larger.

### Fractional atomic coordinates and isotropic or equivalent isotropic displacement parameters (Å^2^)

**Table:**

	*x*	*y*	*z*	*U*_iso_*/*U*_eq_	
Cl1	0.32227 (8)	0.91214 (6)	0.18882 (2)	0.01916 (13)	
Cl2	0.25294 (9)	0.79727 (7)	0.06239 (2)	0.02243 (14)	
Cl3	0.24340 (8)	0.44993 (7)	0.02105 (2)	0.02233 (13)	
Cl4	0.28079 (8)	0.20952 (6)	0.10536 (2)	0.01886 (13)	
Cl5	0.83636 (8)	1.38342 (6)	0.20872 (2)	0.01938 (13)	
Cl6	0.77697 (8)	1.27830 (6)	0.08171 (2)	0.02106 (13)	
Cl7	0.75697 (8)	0.93290 (7)	0.03745 (2)	0.02244 (14)	
Cl8	0.78622 (8)	0.68379 (6)	0.11969 (2)	0.01849 (13)	
F1	0.09797 (18)	0.52932 (15)	0.35163 (5)	0.0272 (3)	
F2	0.1377 (2)	0.44755 (17)	0.45515 (5)	0.0370 (4)	
F3	0.4616 (2)	0.32808 (17)	0.48974 (5)	0.0351 (4)	
F4	1.29401 (19)	1.04373 (16)	0.33909 (5)	0.0290 (3)	
F5	1.4227 (2)	0.98029 (18)	0.44020 (6)	0.0427 (4)	
F6	1.1612 (2)	0.82690 (18)	0.50178 (5)	0.0442 (4)	
O1	0.3982 (2)	0.74947 (18)	0.29461 (6)	0.0233 (4)	
O2	0.3402 (2)	0.23349 (17)	0.23416 (6)	0.0229 (4)	
O3	0.9291 (2)	1.21384 (18)	0.31271 (6)	0.0216 (4)	
O4	0.8648 (2)	0.70099 (18)	0.24803 (6)	0.0257 (4)	
N1	0.3776 (3)	0.4840 (2)	0.27781 (7)	0.0171 (4)	
N2	0.9077 (3)	0.9487 (2)	0.29353 (7)	0.0177 (4)	
C1	0.3739 (3)	0.6333 (3)	0.26336 (9)	0.0176 (5)	
C2	0.3363 (3)	0.6123 (2)	0.20166 (8)	0.0151 (5)	
C3	0.3126 (3)	0.7200 (2)	0.16569 (9)	0.0163 (5)	
C4	0.2817 (3)	0.6680 (3)	0.10912 (8)	0.0168 (5)	
C5	0.2751 (3)	0.5115 (3)	0.09030 (8)	0.0166 (5)	
C6	0.2956 (3)	0.4031 (2)	0.12762 (8)	0.0164 (5)	
C7	0.3259 (3)	0.4568 (3)	0.18312 (8)	0.0160 (5)	
C8	0.3474 (3)	0.3692 (3)	0.23222 (8)	0.0169 (5)	
C9	0.4061 (3)	0.4492 (2)	0.33327 (8)	0.0180 (5)	
C10	0.2611 (3)	0.4700 (3)	0.36825 (9)	0.0208 (5)	
C11	0.2802 (3)	0.4284 (3)	0.42102 (9)	0.0241 (5)	
C12	0.4469 (4)	0.3681 (3)	0.43799 (9)	0.0254 (6)	
C13	0.5958 (4)	0.3496 (3)	0.40436 (9)	0.0251 (6)	
H13	0.7109	0.3090	0.4171	0.030*	
C14	0.5749 (3)	0.3914 (3)	0.35132 (9)	0.0211 (5)	
H14	0.6769	0.3802	0.3274	0.025*	
C15	0.8977 (3)	1.0995 (3)	0.28042 (8)	0.0165 (5)	
C16	0.8468 (3)	1.0809 (2)	0.21917 (8)	0.0155 (5)	
C17	0.8229 (3)	1.1919 (2)	0.18423 (8)	0.0167 (5)	
C18	0.7927 (3)	1.1435 (3)	0.12721 (8)	0.0171 (5)	
C19	0.7833 (3)	0.9880 (3)	0.10714 (8)	0.0170 (5)	
C20	0.8001 (3)	0.8765 (2)	0.14369 (8)	0.0155 (5)	
C21	0.8327 (3)	0.9259 (2)	0.19943 (8)	0.0155 (5)	
C22	0.8675 (3)	0.8368 (3)	0.24746 (8)	0.0169 (5)	
C23	0.9670 (3)	0.9153 (2)	0.34779 (8)	0.0189 (5)	
C24	1.1641 (3)	0.9649 (3)	0.36851 (9)	0.0209 (5)	
C25	1.2298 (4)	0.9340 (3)	0.42078 (9)	0.0257 (6)	
C26	1.0962 (4)	0.8546 (3)	0.45089 (9)	0.0294 (6)	
C27	0.8992 (4)	0.8043 (3)	0.43104 (9)	0.0316 (6)	
H27	0.8088	0.7490	0.4526	0.038*	
C28	0.8346 (4)	0.8359 (3)	0.37858 (9)	0.0254 (5)	
H28	0.6988	0.8026	0.3641	0.031*	

### Atomic displacement parameters (Å^2^)

**Table:**

	*U*^11^	*U*^22^	*U*^33^	*U*^12^	*U*^13^	*U*^23^
Cl1	0.0216 (3)	0.0129 (3)	0.0235 (3)	0.0040 (2)	0.0019 (2)	0.0027 (2)
Cl2	0.0282 (3)	0.0206 (3)	0.0198 (3)	0.0039 (3)	0.0020 (2)	0.0088 (2)
Cl3	0.0299 (3)	0.0228 (3)	0.0139 (2)	0.0036 (3)	0.0013 (2)	0.0017 (2)
Cl4	0.0233 (3)	0.0144 (3)	0.0185 (3)	0.0032 (2)	0.0022 (2)	−0.0004 (2)
Cl5	0.0240 (3)	0.0126 (3)	0.0220 (3)	0.0042 (2)	0.0030 (2)	0.0020 (2)
Cl6	0.0251 (3)	0.0195 (3)	0.0201 (3)	0.0044 (2)	0.0025 (2)	0.0083 (2)
Cl7	0.0308 (3)	0.0228 (3)	0.0136 (3)	0.0044 (3)	0.0018 (2)	0.0017 (2)
Cl8	0.0228 (3)	0.0142 (3)	0.0179 (3)	0.0029 (2)	0.0021 (2)	−0.0008 (2)
F1	0.0239 (7)	0.0282 (8)	0.0303 (7)	0.0102 (6)	0.0008 (6)	0.0002 (6)
F2	0.0414 (9)	0.0446 (10)	0.0289 (8)	0.0109 (8)	0.0184 (7)	0.0044 (7)
F3	0.0549 (10)	0.0372 (10)	0.0147 (7)	0.0081 (8)	0.0041 (7)	0.0084 (6)
F4	0.0300 (8)	0.0265 (8)	0.0295 (8)	−0.0009 (6)	0.0030 (6)	0.0068 (6)
F5	0.0369 (9)	0.0459 (11)	0.0404 (9)	−0.0005 (8)	−0.0177 (7)	0.0088 (8)
F6	0.0619 (11)	0.0490 (11)	0.0223 (8)	0.0097 (9)	−0.0087 (7)	0.0154 (7)
O1	0.0322 (10)	0.0181 (9)	0.0186 (8)	0.0070 (8)	−0.0033 (7)	−0.0026 (7)
O2	0.0354 (10)	0.0141 (9)	0.0192 (8)	0.0041 (7)	0.0022 (7)	0.0026 (6)
O3	0.0298 (9)	0.0161 (9)	0.0179 (8)	0.0053 (7)	0.0002 (7)	−0.0031 (6)
O4	0.0417 (11)	0.0150 (9)	0.0202 (8)	0.0056 (8)	0.0000 (8)	0.0019 (7)
N1	0.0248 (10)	0.0137 (10)	0.0130 (9)	0.0047 (8)	0.0000 (8)	0.0013 (7)
N2	0.0267 (11)	0.0128 (10)	0.0137 (9)	0.0038 (8)	0.0002 (8)	0.0019 (7)
C1	0.0191 (12)	0.0163 (12)	0.0173 (11)	0.0046 (10)	−0.0019 (9)	0.0014 (9)
C2	0.0131 (11)	0.0154 (12)	0.0162 (10)	0.0013 (9)	0.0005 (9)	0.0019 (8)
C3	0.0146 (11)	0.0124 (11)	0.0215 (11)	0.0018 (9)	0.0020 (9)	0.0009 (9)
C4	0.0155 (11)	0.0168 (12)	0.0183 (11)	0.0015 (9)	0.0009 (9)	0.0062 (9)
C5	0.0160 (11)	0.0182 (12)	0.0153 (10)	0.0006 (9)	0.0029 (9)	0.0022 (9)
C6	0.0148 (11)	0.0150 (12)	0.0190 (11)	0.0017 (9)	0.0021 (9)	0.0011 (9)
C7	0.0152 (11)	0.0147 (11)	0.0184 (11)	0.0035 (9)	0.0015 (9)	0.0019 (9)
C8	0.0188 (12)	0.0164 (12)	0.0158 (11)	0.0053 (10)	0.0017 (9)	0.0003 (9)
C9	0.0272 (13)	0.0139 (11)	0.0122 (10)	0.0025 (10)	−0.0002 (9)	0.0008 (8)
C10	0.0229 (13)	0.0174 (12)	0.0215 (11)	0.0047 (10)	−0.0008 (10)	−0.0017 (9)
C11	0.0295 (14)	0.0235 (14)	0.0187 (11)	0.0014 (11)	0.0096 (10)	−0.0013 (10)
C12	0.0417 (16)	0.0206 (13)	0.0130 (11)	0.0006 (11)	0.0019 (11)	0.0039 (9)
C13	0.0313 (14)	0.0260 (14)	0.0192 (12)	0.0095 (11)	−0.0024 (10)	0.0043 (10)
C14	0.0255 (13)	0.0202 (13)	0.0188 (11)	0.0057 (10)	0.0048 (10)	0.0022 (9)
C15	0.0168 (11)	0.0150 (12)	0.0182 (11)	0.0039 (9)	0.0013 (9)	0.0024 (9)
C16	0.0144 (11)	0.0167 (12)	0.0149 (10)	0.0025 (9)	0.0007 (9)	0.0005 (9)
C17	0.0165 (12)	0.0133 (11)	0.0202 (11)	0.0020 (9)	0.0026 (9)	0.0019 (9)
C18	0.0154 (11)	0.0173 (12)	0.0192 (11)	0.0029 (9)	0.0015 (9)	0.0050 (9)
C19	0.0165 (11)	0.0203 (12)	0.0140 (10)	0.0015 (9)	0.0019 (9)	0.0027 (9)
C20	0.0123 (11)	0.0151 (12)	0.0184 (11)	0.0013 (9)	0.0009 (9)	0.0004 (9)
C21	0.0178 (12)	0.0134 (11)	0.0158 (10)	0.0040 (9)	0.0018 (9)	0.0014 (8)
C22	0.0192 (12)	0.0150 (12)	0.0162 (10)	0.0031 (9)	0.0014 (9)	0.0004 (9)
C23	0.0279 (13)	0.0156 (12)	0.0137 (10)	0.0064 (10)	−0.0008 (10)	0.0015 (9)
C24	0.0251 (13)	0.0168 (12)	0.0210 (11)	0.0028 (10)	0.0032 (10)	0.0034 (9)
C25	0.0273 (14)	0.0239 (14)	0.0238 (12)	0.0047 (11)	−0.0077 (11)	0.0006 (10)
C26	0.0438 (17)	0.0286 (15)	0.0164 (11)	0.0093 (13)	−0.0042 (11)	0.0055 (10)
C27	0.0409 (16)	0.0349 (16)	0.0209 (12)	0.0054 (13)	0.0058 (12)	0.0112 (11)
C28	0.0300 (14)	0.0258 (14)	0.0197 (12)	0.0021 (11)	0.0016 (10)	0.0030 (10)

### Geometric parameters (Å, °)

**Table:**

Cl1—C3	1.719 (2)	C4—C5	1.401 (3)
Cl2—C4	1.714 (2)	C5—C6	1.401 (3)
Cl3—C5	1.714 (2)	C6—C7	1.382 (3)
Cl4—C6	1.722 (2)	C7—C8	1.503 (3)
Cl5—C17	1.718 (2)	C9—C10	1.375 (3)
Cl6—C18	1.717 (2)	C9—C14	1.381 (3)
Cl7—C19	1.715 (2)	C10—C11	1.380 (3)
Cl8—C20	1.725 (2)	C11—C12	1.373 (3)
F1—C10	1.344 (2)	C12—C13	1.370 (3)
F2—C11	1.344 (2)	C13—C14	1.388 (3)
F3—C12	1.350 (2)	C13—H13	0.9500
F4—C24	1.337 (2)	C14—H14	0.9500
F5—C25	1.341 (3)	C15—C16	1.499 (3)
F6—C26	1.347 (2)	C16—C17	1.382 (3)
O1—C1	1.196 (2)	C16—C21	1.387 (3)
O2—C8	1.194 (2)	C17—C18	1.408 (3)
O3—C15	1.197 (2)	C18—C19	1.398 (3)
O4—C22	1.195 (2)	C19—C20	1.404 (3)
N1—C1	1.399 (3)	C20—C21	1.380 (3)
N1—C8	1.409 (2)	C21—C22	1.501 (3)
N1—C9	1.427 (2)	C23—C28	1.371 (3)
N2—C22	1.402 (2)	C23—C24	1.378 (3)
N2—C15	1.407 (3)	C24—C25	1.389 (3)
N2—C23	1.430 (2)	C25—C26	1.366 (3)
C1—C2	1.500 (3)	C26—C27	1.374 (3)
C2—C3	1.376 (3)	C27—C28	1.393 (3)
C2—C7	1.387 (3)	C27—H27	0.9500
C3—C4	1.404 (3)	C28—H28	0.9500
			
C1—N1—C8	113.57 (17)	C9—C14—C13	120.1 (2)
C1—N1—C9	123.83 (18)	C9—C14—H14	120.0
C8—N1—C9	122.60 (18)	C13—C14—H14	120.0
C22—N2—C15	113.18 (17)	O3—C15—N2	125.24 (19)
C22—N2—C23	123.55 (18)	O3—C15—C16	130.1 (2)
C15—N2—C23	123.13 (18)	N2—C15—C16	104.68 (17)
O1—C1—N1	125.92 (19)	C17—C16—C21	121.68 (19)
O1—C1—C2	129.4 (2)	C17—C16—C15	129.5 (2)
N1—C1—C2	104.68 (17)	C21—C16—C15	108.75 (18)
C3—C2—C7	121.54 (19)	C16—C17—C18	117.6 (2)
C3—C2—C1	129.7 (2)	C16—C17—Cl5	121.73 (16)
C7—C2—C1	108.72 (18)	C18—C17—Cl5	120.65 (16)
C2—C3—C4	117.8 (2)	C19—C18—C17	120.79 (19)
C2—C3—Cl1	121.46 (17)	C19—C18—Cl6	119.63 (16)
C4—C3—Cl1	120.77 (16)	C17—C18—Cl6	119.54 (17)
C5—C4—C3	120.72 (19)	C18—C19—C20	120.45 (19)
C5—C4—Cl2	119.49 (16)	C18—C19—Cl7	119.97 (16)
C3—C4—Cl2	119.79 (17)	C20—C19—Cl7	119.56 (17)
C4—C5—C6	120.68 (19)	C21—C20—C19	118.1 (2)
C4—C5—Cl3	120.40 (16)	C21—C20—Cl8	120.78 (16)
C6—C5—Cl3	118.92 (17)	C19—C20—Cl8	121.09 (16)
C7—C6—C5	117.6 (2)	C20—C21—C16	121.3 (2)
C7—C6—Cl4	121.10 (17)	C20—C21—C22	130.2 (2)
C5—C6—Cl4	121.33 (17)	C16—C21—C22	108.44 (18)
C6—C7—C2	121.7 (2)	O4—C22—N2	125.79 (19)
C6—C7—C8	129.7 (2)	O4—C22—C21	129.3 (2)
C2—C7—C8	108.60 (18)	N2—C22—C21	104.91 (18)
O2—C8—N1	125.93 (19)	C28—C23—C24	120.4 (2)
O2—C8—C7	129.7 (2)	C28—C23—N2	121.9 (2)
N1—C8—C7	104.38 (18)	C24—C23—N2	117.7 (2)
C10—C9—C14	120.17 (19)	F4—C24—C23	120.94 (19)
C10—C9—N1	119.27 (19)	F4—C24—C25	119.1 (2)
C14—C9—N1	120.53 (19)	C23—C24—C25	120.0 (2)
F1—C10—C9	120.85 (19)	F5—C25—C26	121.5 (2)
F1—C10—C11	119.0 (2)	F5—C25—C24	119.6 (2)
C9—C10—C11	120.2 (2)	C26—C25—C24	118.9 (2)
F2—C11—C12	120.8 (2)	F6—C26—C25	118.4 (2)
F2—C11—C10	120.3 (2)	F6—C26—C27	119.6 (2)
C12—C11—C10	118.9 (2)	C25—C26—C27	122.0 (2)
F3—C12—C13	120.3 (2)	C26—C27—C28	118.6 (2)
F3—C12—C11	117.7 (2)	C26—C27—H27	120.7
C13—C12—C11	122.0 (2)	C28—C27—H27	120.7
C12—C13—C14	118.6 (2)	C23—C28—C27	120.0 (2)
C12—C13—H13	120.7	C23—C28—H28	120.0
C14—C13—H13	120.7	C27—C28—H28	120.0
			
C8—N1—C1—O1	−179.0 (2)	C22—N2—C15—O3	−178.8 (2)
C9—N1—C1—O1	1.9 (4)	C23—N2—C15—O3	−3.1 (4)
C8—N1—C1—C2	0.2 (2)	C22—N2—C15—C16	−0.4 (2)
C9—N1—C1—C2	−178.89 (19)	C23—N2—C15—C16	175.38 (19)
O1—C1—C2—C3	−2.9 (4)	O3—C15—C16—C17	−0.1 (4)
N1—C1—C2—C3	177.9 (2)	N2—C15—C16—C17	−178.4 (2)
O1—C1—C2—C7	177.5 (2)	O3—C15—C16—C21	177.4 (2)
N1—C1—C2—C7	−1.7 (2)	N2—C15—C16—C21	−0.9 (2)
C7—C2—C3—C4	−1.4 (3)	C21—C16—C17—C18	−2.9 (3)
C1—C2—C3—C4	179.1 (2)	C15—C16—C17—C18	174.3 (2)
C7—C2—C3—Cl1	179.12 (16)	C21—C16—C17—Cl5	178.95 (16)
C1—C2—C3—Cl1	−0.4 (3)	C15—C16—C17—Cl5	−3.8 (3)
C2—C3—C4—C5	0.1 (3)	C16—C17—C18—C19	1.3 (3)
Cl1—C3—C4—C5	179.60 (16)	Cl5—C17—C18—C19	179.47 (16)
C2—C3—C4—Cl2	−179.57 (16)	C16—C17—C18—Cl6	−176.17 (16)
Cl1—C3—C4—Cl2	−0.1 (3)	Cl5—C17—C18—Cl6	2.0 (3)
C3—C4—C5—C6	1.2 (3)	C17—C18—C19—C20	1.2 (3)
Cl2—C4—C5—C6	−179.15 (16)	Cl6—C18—C19—C20	178.69 (16)
C3—C4—C5—Cl3	−178.42 (16)	C17—C18—C19—Cl7	−177.29 (16)
Cl2—C4—C5—Cl3	1.3 (3)	Cl6—C18—C19—Cl7	0.2 (3)
C4—C5—C6—C7	−1.1 (3)	C18—C19—C20—C21	−2.1 (3)
Cl3—C5—C6—C7	178.44 (15)	Cl7—C19—C20—C21	176.36 (16)
C4—C5—C6—Cl4	178.15 (16)	C18—C19—C20—Cl8	179.31 (16)
Cl3—C5—C6—Cl4	−2.3 (3)	Cl7—C19—C20—Cl8	−2.2 (3)
C5—C6—C7—C2	−0.1 (3)	C19—C20—C21—C16	0.6 (3)
Cl4—C6—C7—C2	−179.42 (16)	Cl8—C20—C21—C16	179.13 (16)
C5—C6—C7—C8	178.2 (2)	C19—C20—C21—C22	−176.3 (2)
Cl4—C6—C7—C8	−1.1 (3)	Cl8—C20—C21—C22	2.2 (3)
C3—C2—C7—C6	1.4 (3)	C17—C16—C21—C20	2.0 (3)
C1—C2—C7—C6	−178.94 (19)	C15—C16—C21—C20	−175.73 (19)
C3—C2—C7—C8	−177.2 (2)	C17—C16—C21—C22	179.53 (19)
C1—C2—C7—C8	2.4 (2)	C15—C16—C21—C22	1.8 (2)
C1—N1—C8—O2	−178.4 (2)	C15—N2—C22—O4	−179.2 (2)
C9—N1—C8—O2	0.8 (4)	C23—N2—C22—O4	5.1 (4)
C1—N1—C8—C7	1.1 (2)	C15—N2—C22—C21	1.4 (2)
C9—N1—C8—C7	−179.71 (18)	C23—N2—C22—C21	−174.33 (19)
C6—C7—C8—O2	−1.2 (4)	C20—C21—C22—O4	−4.1 (4)
C2—C7—C8—O2	177.3 (2)	C16—C21—C22—O4	178.7 (2)
C6—C7—C8—N1	179.3 (2)	C20—C21—C22—N2	175.3 (2)
C2—C7—C8—N1	−2.2 (2)	C16—C21—C22—N2	−2.0 (2)
C1—N1—C9—C10	66.6 (3)	C22—N2—C23—C28	−72.5 (3)
C8—N1—C9—C10	−112.4 (2)	C15—N2—C23—C28	112.2 (3)
C1—N1—C9—C14	−115.1 (2)	C22—N2—C23—C24	107.1 (2)
C8—N1—C9—C14	65.9 (3)	C15—N2—C23—C24	−68.2 (3)
C14—C9—C10—F1	178.6 (2)	C28—C23—C24—F4	−179.3 (2)
N1—C9—C10—F1	−3.1 (3)	N2—C23—C24—F4	1.1 (3)
C14—C9—C10—C11	−2.1 (4)	C28—C23—C24—C25	0.2 (3)
N1—C9—C10—C11	176.2 (2)	N2—C23—C24—C25	−179.4 (2)
F1—C10—C11—F2	−0.2 (4)	F4—C24—C25—F5	−1.6 (3)
C9—C10—C11—F2	−179.5 (2)	C23—C24—C25—F5	178.8 (2)
F1—C10—C11—C12	−179.9 (2)	F4—C24—C25—C26	179.4 (2)
C9—C10—C11—C12	0.8 (4)	C23—C24—C25—C26	−0.1 (4)
F2—C11—C12—F3	0.4 (4)	F5—C25—C26—F6	1.8 (4)
C10—C11—C12—F3	−179.8 (2)	C24—C25—C26—F6	−179.3 (2)
F2—C11—C12—C13	−179.0 (2)	F5—C25—C26—C27	−178.8 (2)
C10—C11—C12—C13	0.7 (4)	C24—C25—C26—C27	0.1 (4)
F3—C12—C13—C14	179.7 (2)	F6—C26—C27—C28	179.2 (2)
C11—C12—C13—C14	−0.9 (4)	C25—C26—C27—C28	−0.2 (4)
C10—C9—C14—C13	2.0 (4)	C24—C23—C28—C27	−0.3 (3)
N1—C9—C14—C13	−176.3 (2)	N2—C23—C28—C27	179.3 (2)
C12—C13—C14—C9	−0.5 (4)	C26—C27—C28—C23	0.3 (4)

### Hydrogen-bond geometry (Å, °)

**Table:**

*D*—H···*A*	*D*—H	H···*A*	*D*···*A*	*D*—H···*A*
C14—H14···O3^i^	0.95	2.44	3.210 (3)	138.
C27—H27···F3^ii^	0.95	2.45	3.379 (3)	165.
C28—H28···O1	0.95	2.50	3.403 (3)	158.

Symmetry codes: (i) *x*, *y*−1, *z*; (ii) −*x*+1, −*y*+1, −*z*+1.
